# Binding Analysis of Functionalized Multimode Optical-Fiber Sandwich-like Structure with Organic Polymer and Its Sensing Application for Humidity and Breath Monitoring

**DOI:** 10.3390/bios11090324

**Published:** 2021-09-08

**Authors:** Daniel Jauregui-Vazquez, Paulina Lozano-Sotomayor, Jorge Emmanuel Mejía-Benavides, Erik Díaz-Cervantes

**Affiliations:** 1 Departamento de Ingeniería Electrónica, División de Ingenierías Campus Irapuato Salamanca, Universidad de Guanajuato, Carretera Salamanca-Valle de Santiago Km 3.5 + 1.8 Km, Salamanca, Guanajuato 36885, Mexico; jaureguid@ugto.mx; 2 Centro Interdisciplinario del Noreste (CINUG), Universidad de Guanajuato, Tierra Blanca, Guanajuato 37975, Mexico; 3 Laboratorio Nacional de Caracterización de Propiedades Fisicoquímicas y Estructura Molecular, Departamento de Química, Universidad de Guanajuato, Guanajuato 36050, Mexico; paulinalozano7@gmail.com; 4 Departamento de Enfermería y Obstetricia, Centro Interdisciplinario del Noreste (CINUG), Universidad de Guanajuato, Tierra Blanca, Guanajuato 37975, Mexico; je.mejiabenavides@ugto.mx; 5 Departamento de Alimentos, Centro Interdisciplinario del Noreste (CINUG), Universidad de Guanajuato, Tierra Blanca, Guanajuato 37975, Mexico

**Keywords:** optical fiber, APTES–alginate, DFT, breath monitoring

## Abstract

In recent years, the chemical modification of optical fibers (OFs) has facilitated the manufacture of sensors because OFs can identify several analytes present in aqueous solutions or gas phases. Nevertheless, it is imperative better to understand the chemical interactions in this molecular system to generate low-cost and efficient sensors. This work presents a theoretical and experimental study of organic polymeric functionalized OF structures and proposes a cost-effective alternative to monitor breathing and humidity. The device is based on silicon optical fibers functionalized with (3-Aminopropyl) triethoxysilane (APTES) and alginate. The theoretical analysis is carried out to validate the activation of the silicon dioxide fiber surface; moreover, the APTES–alginate layer is discussed. The computational simulation suggests that water can be absorbed by alginate, specifically by the calcium atom linked to the carboxylic acid group of the alginate. The analysis also demonstrates a higher electrostatic interaction between the water and the OF–APTES–alginate system; this interaction alters the optical fiber activated surface’s refractive index, resulting in transmission power variation. The humidity analysis shows a sensitivity of 3.1288 mV/RH, a time response close to 25 s, and a recovery time around 8 s. These results were achieved in the range of 50 to 95% RH. Moreover, the recovery and response time allow the human breath to be studied. The proposed mechanism or device is competitive with prior works, and the components involved made this sensor a cost-effective alternative for medical applications.

## 1. Introduction

Human breathing monitoring is nowadays more relevant due to the COVID-19 pandemic. This parameter is related to humidity detection. There is global interest in the development of new devises that make it possible to measure humidity using versatile, economic, and optimized mechanisms.

One alternative is humidity optical fiber sensors, which require the functionalization of optical fibers (OFs). This procedure modifies the fiber optic surface and generates a sensitive layer to water absorption; this route alters the refractive index layer [1]. The selective layer can be designed to promote sensors applied to several science fields and highlight biological and biochemical applications [2,3]. In this regard, the state-of-the-art method uses functionalized OF (f-OF) with small molecules, such as organic acids [4], representing some biomolecules such as DNA [5].

Here, bridge molecules are used to connect a selective molecule to the OF. Then, the OF needs to be activated with some labile functional groups that stimulate chemical functionalization. The typical bridge molecule used to modify the silicon derivatives [6] is (3-Aminopropyl) triethoxysilane (APTES), employed to generate an available amine group on the tip of the whole system.

Moreover, the generation of f-OF has focused on coating the OF with some biopolymers [7,8,9]. Here, chitosan and all-fiber Fabry–Perot cavities have been demonstrated as a versatile combination to detect humidity and monitor breath [10,11,12]. Another non-typical biopolymer used for humidity sensing is alginate, a derivative of natural complex sugars [13].

These materials, as others, have been used for humidity sensing based on phase and intensity modulation [14,15,16]. Despite that humidity sensors based on phase modulation offer immunity to power fluctuations [17,18], its demodulation process is intricate. Furthermore, intensity humidity detection can be implemented at a low cost [19,20,21].

Even with the efforts to propose and study new materials, it is still necessary to understand, predict, and validate the working operation. The present work proposes a binding study of manufactured optical fiber humidity sensors based on APTES and alginate. Here, it is possible to explain the molecular interactions that promote water adsorption in our proposed system.

## 2. Materials and Methods

### 2.1. Optical Fiber Functionalization

**Figure 1:**
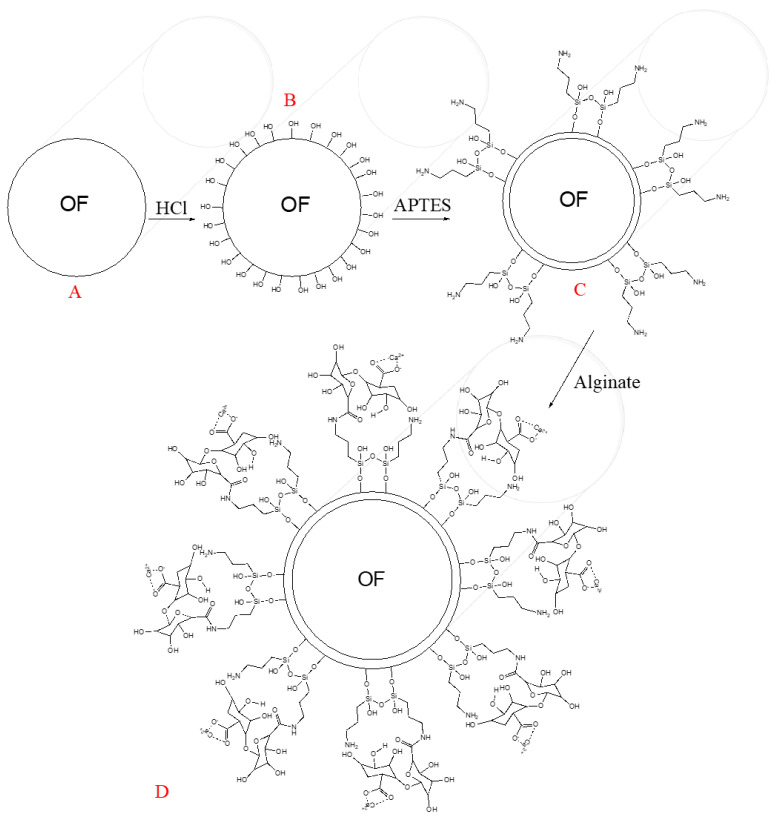
Scheme of functionalization of optical fibers with alginate. (**A**) silicon dioxide optical fiber, (**B**) hydroxyl groups formation, (**C**) APTES integration, and (**D**) calcium alginate reaction.

To active the silicon dioxide of the optical fibers (OFs), which is depicted in the transversal view in Figure 1A, it was soaked in HCl [5 M] for 30 min, which promotes the generation of hydroxyl groups (see Figure 1B) on the OF surface, as Zhang and co-workers explain [5]. Afterwards, the next step was rinsing the OF surface with deionized water and air drying. Figure 1C shows the second step of the OF chemical modification. The APTES reacts with the OF surface’s hydroxyl groups, promoting silanol group formation with some amine groups on the whole system’s tip, producing the so-called OF–APTES. To promote the above step, it was necessary to immerse the active optical fiber into APTES (10% acid alcohol solution) for 50 min. Once the formation of OF–APTES was promoted, it was incubated in calcium alginate solution for two hours, following the methodology described by [22], generating the system shown in Figure 1D.

### 2.2. In Silico Molecular Interactions of the Functionalized OF

The chemical interactions of functionalized optical fibers have been studied in the present work through a finite molecular model of the OF, based on the expanded unitary cell of the solid state of SiO_2_. The APTES and the alginate structures were modeled considering only two monomers of each.

The whole molecules were modeled using Avogadro software [23]. The optimization process was carried out through the formalism of the density functional theory [24,25,26,27,28], specifically using Perdew, Burke, and Ernzerhof [29]. The GGA functional, the pseudopotential of Los Alamos National Laboratory (LANL2DZ) for the calcium atom, as well as the Pople 6-31G(d,p) basis [30] were set for the other kinds of elements, through the Gaussian 09 (G09) [31] package.

## 3. Results and Discussion

### 3.1. Molecular Interactions of the Functionalized OF

Following the method mentioned above, we generated an alginate functionalized optical fiber, which was analyzed and validated using scanning electron microscopy (SEM) and in silico assays.

**Figure 2:**
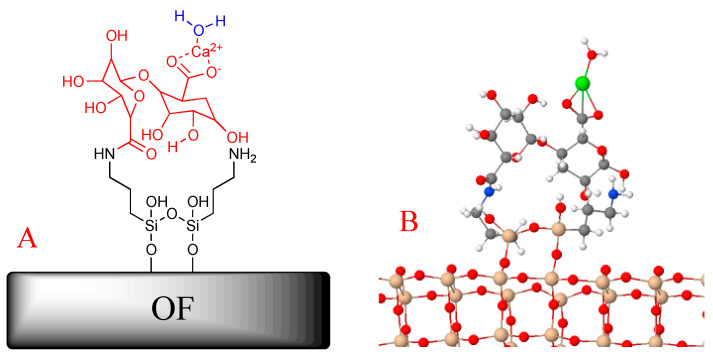
(**A**) 2D and (**B**) 3D models of the functionalized optical fiber.

**Figure 3:**
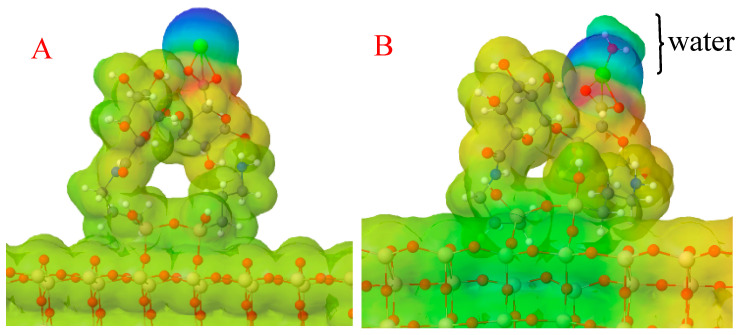
Molecular electrostatic potential surface for the (**A**) OF–APTES–alginate and the (**B**) OF–APTES–alginate–water systems. Blue surfaces represent the zones with lower electronic density, red surfaces the sites with higher electronic density, and green the middle point.

The final chemical binding system is depicted in Figure 2A (red structure); the APTES (black lines in Figure 2A) acts as a bridge between the OF and the biopolymer. In agreement with the obtained model (see Figure 3B), the way to link water to the OF–APTES–alginate system is via the calcium located on the end of the proposed chemical configuration.

To understand the water adsorption on the OF–APTES–alginate system, a finite model shown in Figure 2B was used. The subsequent reaction was evaluated to obtain the adsorption energy through Equation (1).

OF-APTES-Alginate + H_2_O → OF-APTES-Alginate-H_2_O E_ads_ = E_OF-APTES-Alginate-H2O_ − (E_OF-APTES-Alginate_ + E_H2O_) (1)

The computed adsorption energy of water on the OF–APTES–alginate surface was −9.85 eV, which indicates that the reaction presented an exergonic behavior, promoting the adhesion of water on the modified OF. Furthermore, to understand the water’s binding mode on the OF–APTES–alginate system, a molecular electrostatic potential surface for this system was calculated (see Figure 3). A polarization effect of the OF–APTES–alginate system (Figure 3A) is evident due to the cationic behavior of the calcium atom (green sphere), which means a lower electronic density site where the calcium is found.

The lower concentration of electronic density causes a positive partial charge in the calcium site, which can attract the atom with higher electronegativity in the water molecule, the oxygen, as can be observed in Figure 3B. Note that the water on the OF–APTES–alginate system stabilizes the OF’s electronic density distribution, but the tip remains polarized.

**Figure 4:**
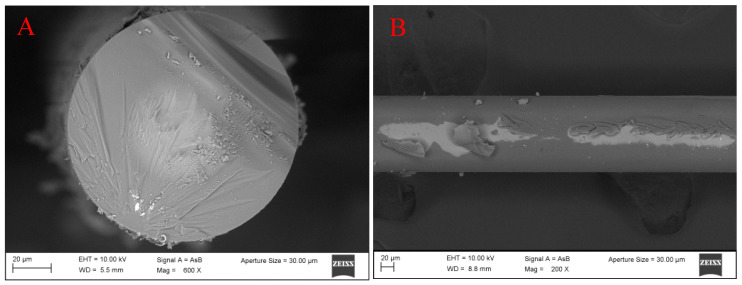
(**A**) Frontal and (**B**) lateral views of the treated NCF.

The functionalization of the OF was validated using SEM. The image shows a nanocoating around the optical fiber of a few nanometers, which confirms the APTES–alginate adhesion to the NCF surface using the above method of functionalization, see Figure 4.

### 3.2. Principle Operation of the Proposed Optical Fiber Sandwich-like Structure

Once the binding analysis validated the proposed chemical process, a silica No-core Fiber (NCF) was functionalized by the method described above. With the purpose to excite an evanescent field, this fiber was spliced between two segments of Multimode Fiber (MMF); here, the MMF had a core radius of 62.5/125 μm, and the NCF had a total diameter of 125 μm. This optical fiber structure was achieved by an arc splicer, Fujikura FSM-17s, using a multimode program. The total length of the NCF was around L = 70 mm, and this was the sensitive region of our proposed device.

**Figure 5:**
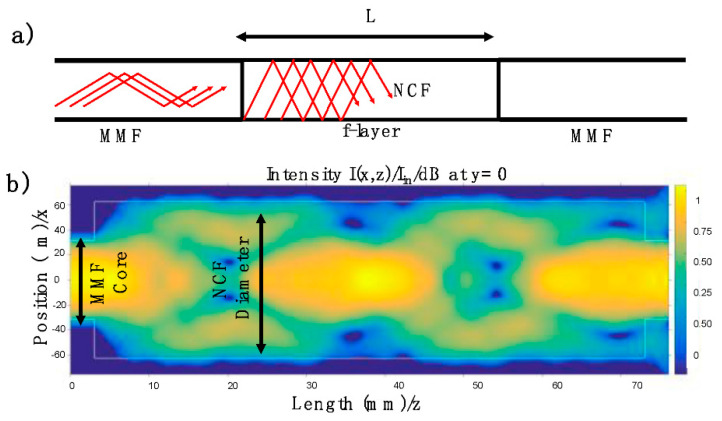
(**a**) Configuration of the optical fiber structure composed by an MMF and NCF; the active region is represented by the f-layer. (**b**) Intensity light propagation distribution through the NCF by using a launch wavelength at 522 nm.

The schematic optical fiber structure is depicted in Figure 5a. It is important to stress that the optical fiber structure was fabricated, and then it was treated by the proposed method. When light travels through the MMF, several modes are excited. Considering the numerical aperture of the MMF (N.A. = 0.22), its core diameter (62.5 μm), and an arbitrary propagation wavelength centered at 522 nm, more than 1300 modes can be presented into the MMF. All this energy arrives at the NCF; then different optical paths are exited. By using the CodeSeeder-BeamLab the energy distribution is studied. Here, the electric field’s intensity propagation distribution is computed for an arbitrary wavelength at 522 nm. As can be appreciated in Figure 5b, the energy interacts with the surrounding medium around the NCF. It is essential to mention that the analysis was carried out without considering the refractive index of the f-layer, and the purpose of this analysis was to observe the intensity distribution. It is important to stress that the transmitted light through the NCF can be expressed by [32,33]:

(2) $$T\left(\lambda \right)=10\times \log\left({\left|\overset{M}{\underset{m=1}{\sum }}c_{0m}^{2}e^{j\beta_{0m}z}\right|}^{2}\right)$$

where the propagation constant ($\beta_{0m}$) and the excitation coefficient ($c_{0m}$) are related to the high-order modes excited at the NCF; furthermore, m represents the numbers of modes excited. It is important to stress that the surrounding media acts as the cladding in the NCF. Then, the output energy will depend on the interaction between the high-order modes exited and the surrounding media. Here, $c_{0m}$ and $\beta_{0m}$ are affected by the refractive index at the f-layer. It is necessary to mention that the NCF provides a multimode interference effect and increases the evanescent field, Figure 5b [33].

### 3.3. Experimental Setup

Using a broadband light source, a significant energy interaction between the NCF and the f-layer can be expected. Considering the launched energy, represented by the ray trajectories shown in Figure 5a, different effective refractive indices were presented. Their values were close to the refractive index of the silica material (1.4605). These modes interacted with the f-layer, composed by APTES with a refractive index of 1.4225 and alginate (1.3310). It can be inferred that the refractive index of the f-layer will be close to the APTES, and once the alginate reaches any surrounding water molecule, the f-layer refractive index tends to the alginate refractive index [3]. Then, as the refractive of the f-layer ($n_{2}$) is modified, the deep penetration field is altered; as a consequence, the total intensity transmitted is changed.

Hence, when the refractive index of the f-layer is similar to the effective refractive index of the mode involved, the energy outside the NCF will be significant; as a result, the transmitted power output decreases. In contrast, where the f-layer refractive index is below the effective refractive index of the modes involved (water interaction), the deep penetration film decreases; thus, the total transmission power increases. The refractive index alteration can be correlated with the computational simulation results. Here, the charge balance in the molecular systems varied due to the water presence, modifying the system’s electronic structure, and promoting change in the transmitted power. Moreover, the higher interaction between water and OF–APTES–alginate promotes a rupture. Hence, the OF and APTES–alginate chemical interactions are re-stabilized.

**Figure 6:**
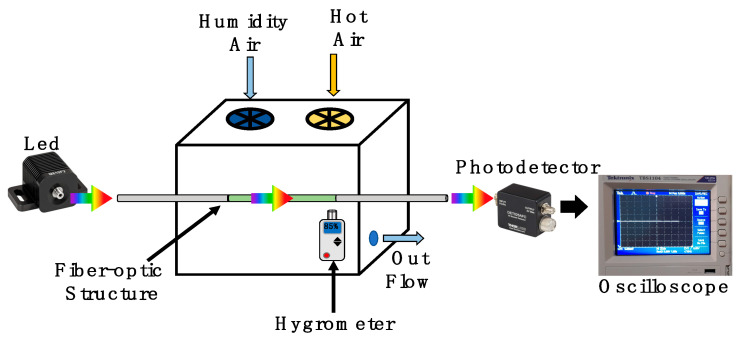
A sketch of the experimental setup used to validate the humidity response of the functionalized optical fiber sandwich structure.

The schematic setup presented in Figure 6 was implemented to analyze the humidity application. Here, a broadband fiber-coupled LED (MBB1F1) was used as an input light signal. The LED provides a broad visible spectrum from 480 nm to 850 nm, with a central wavelength at 522 nm. In our analysis, the LED operated with a current driver of 250 mA and maximal output power 132μW. This energy was launched to the functionalized optical fiber structure. Here, the optical fiber structure was suspended into a cube box chamber with a 5-inch edge. The relative humidity was controlled by a humidifier and hot-air station, both of them with a control timer. Into the chamber, a hygrometer UT33 was set close to the optical fiber structure to monitor its surrounding relative humidity and temperature. Moreover, the chamber had a valve to control the output flow. To monitor the power fluctuations, a high-speed coupled fiber photodetector (DET025AFC) was used. Its operation wavelength range was 400 to 1100 nm. Considering the wavelength-centered peak of the LED and the limit of its bandwidth, the quantum efficiency can be estimated at 98.34% at 580 nm and 66.8% at 850 nm. It is important to mention that the maximal responsivity of the photodetector is 0.46 A/W. This element was set at the end of the functionalized optical fiber structure. Moreover, the transmission was monitored by an oscilloscope (TBS1104).

### 3.4. Humidity and Breath Monitoring Analyses

**Figure 7:**
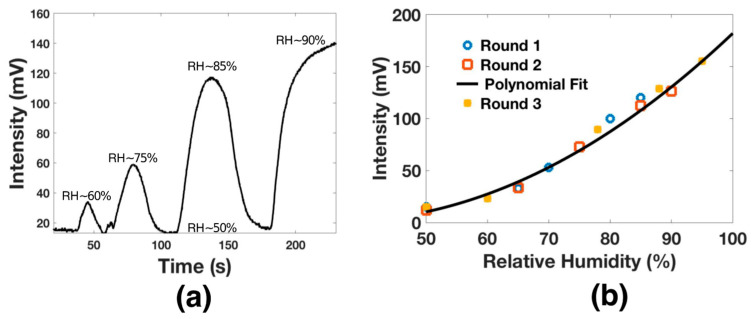
(**a**) The voltage output response of functionalized optical fiber for different RH. (**b**) Relative humidity sensitivity analysis.

The humidity analysis was carried out by setting the oscilloscope at 20 mV/DIV and 5 s. Then, the humidity was increased by specific intervals; it is essential to mention that the chamber had a relative humidity of 50% when increasing the humidity and exhibited an average temperature of 25 °C. Here, the humidifier was turned on for 10 s, and then it was turned off; thus, the relative humidity (RH) increased to 60%. Consequently, the voltage signal increased 10 mV. Then, the valve was opened and closed, and the process was repeated until the hygrometer detected an RH close to 75%. Here, the output signal was 60 mV. The process was repeated until the hygrometer detected a high RH around 90%. Here, the maximal output voltage was 140 mV. The relative humidity response is shown in Figure 7a.

The analysis was performed several times, and the results are shown in Figure 7b. As can be appreciated, the proposed functionalized optical fiber structure presented a polynomial response with maximal sensitivity of 3.1288 mV/RH. This sensitivity was achieved over the dynamic range from 50% RH to 95% RH. The polynomial response can be expressed by x^2^ − 8x + 210; this response presents an adjusted R-squared (R^2^) of 0.9941. It is important to recall that this sensitivity is competitive with some prior works [34,35,36] and can be improved by applying some f-layers [34]; however, this process exposes the structure and reduce the repeatability.

**Figure 8:**
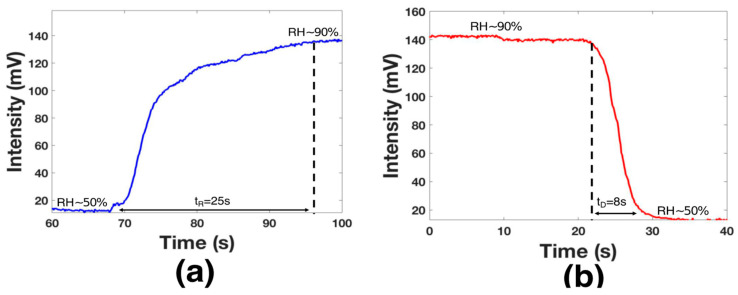
Analysis of the time response (**a**) and decay time (**b**).

**Figure 9:**
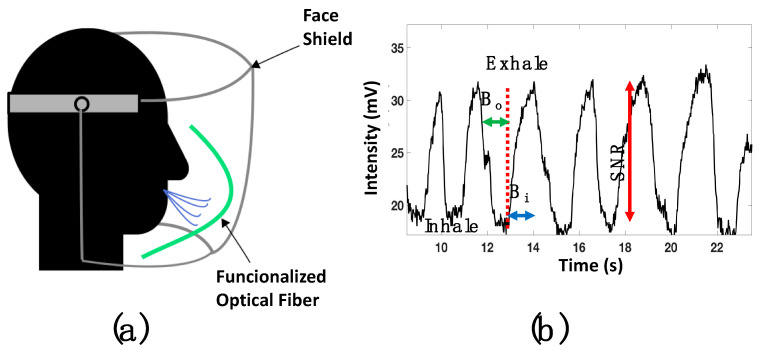
(**a**) Sketch of the human breath monitoring analysis; (**b**) voltage response of human breath monitoring using a face shield.

The time response (tR) was analyzed by activating the humidifier until the chamber had an RH = 90% (see Figure 8a); here, the transmitted voltage power was from 14 mv to 140 mV. As can be observed, the voltage variation took 25 s until the device was saturated; however, this value was limited by the chamber design. The decay time (tD) was analyzed by using the hot-air station and by opening the chamber valve (see Figure 8a). The functionalized layer needed 8 s to return to the initial point; this time was lower than that in some recent works [37,38]. It is essential to mention that the sensor did not present a response for humidity below 50%. Considering the tD and the dynamic range results, the proposed device can monitor human breath. Hence, the functionalized optical fiber was fixed using special glue in the middle of the mask (see Figure 9a). A healthy human male provided respiration; at this point, the distance between the mouth and the functionalized optical fiber was 20 mm. The presented results are competitive with prior fiber optic humidity sensors. Here, the terms tR and tD are considered (see Table 1); furthermore, the functionalization process is a cost-effective alternative.

Once the optical fiber was set into the face shield, a DC reference level (17 mv) was set into the oscilloscope. It is important to consider that the functionalized fiber is bent; as a result, the macro-bending generates a power decrement. The respiratory rate was analyzed by setting the oscilloscope parameters (amplitude and time) at 5 mV and 5 s. Then, the human breath was monitored, and the response is presented in Figure 9b. The respiratory process is described by the dips, representing inhalation, and the peaks that show the exhalation. This signal had a good signal-to-noise ratio (SNR) around 12 dB.

**Figure 10:**
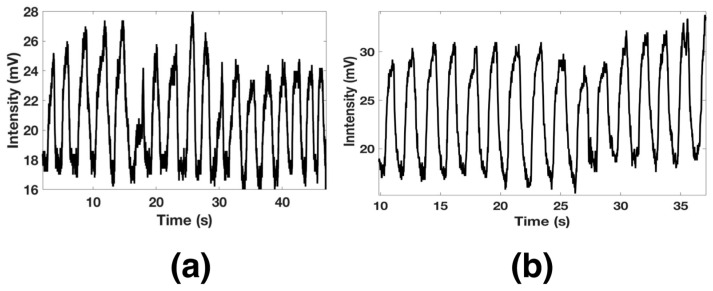
Slow (**a**) and fast (**b**) human breath monitoring.

Furthermore, the functionalized optical fiber allows us to detect a long breath-out (Bo) duration of 1.12 s and minimal breath-in (Bi) around 600 ms. The pattern of a regular human breath is shown in Figure 10a, where a rate of 18 breaths per minute was observed. The functionalized optical fiber was also proved for fast breath monitoring. After a short exercise time, the respiratory rate was measured and is shown in Figure 10b. It can be observed that the sensors exhibited good response and could detect 15 breaths in 30 s.

## 4. Conclusions

Alginate-functionalized optical fiber was theoretically and experimentally validated for humidity and human breath monitoring. The theoretical analysis was performed to understand and validate the binding analysis between alginate and APTES on a silicon dioxide optical fiber surface. The study reveals that water molecules can be easily attracted by the calcium located at the end of the structure. Furthermore, the adsorption energy of water on the OF–APTES–alginate surface was computed close to −9.85 eV, and then an exergonic response can promote the adhesion of water on the modified OF surface. Besides, the in silico assay indicates a polarization effect of the OF–APTES–alginate system; this is related to the calcium atom’s cationic behavior. Consequently, a lower electronic density site can be expected where the calcium is located. Then, water modifies the system’s electronic structure and promotes a change in the transmitted power. This molecule adhesion is also related to the refractive index alteration. We would like to draw attention to the optical fiber sandwich-like structure (composed of Non-core optical and Multi-mode optical) that we fabricated, which promoted the interaction between light and the functionalized layer.

The total length of the Non-core optical fiber was 70 mm. This material was treated by the chemical process described above, and the humidity and breath monitoring analysis were carried out. The humidity analysis indicated a sensitivity of 3.1288 mV/RH in the range between 50% to 90% RH. Furthermore, the time response was close to 25 s, and the recovery time was around 8 s. These results made this functionalized optical fiber attractive for human breath monitoring. Then, this experimental validation was carried out by setting the fiber on a face shield; here, it was possible to detect human breath monitoring with a signal-to-noise ratio of 12 dB. Moreover, a long breath out of 1.12 s and minimal breath in of around 600 ms were monitored. The proposed structure is competitive with prior works, and the used materials made this sensor a cost-effective alternative for medical applications.

## Figures and Tables

**Table 1:** Comparative time response.

Functionalizing Material	tR/tD (s)	Reference
SiO_2_-TiO_2_	50/25	[21]
Polymethyl methacrylate	168/27	[39]
Silica sol-gel	180/30	[40]
Sulfonated polyimides	240/60	[41]
Sulfonated polystyrene	300/30	[42]
TiO_2_	190/100	[43]
Alginate–APTES	25/8	This work
